# Inborn errors in the metabolism of glutathione

**DOI:** 10.1186/1750-1172-2-16

**Published:** 2007-03-30

**Authors:** Ellinor Ristoff, Agne Larsson

**Affiliations:** 1 Karolinska Institute, Department of Pediatrics, Karolinska University Hospital Huddinge, SE-141 86 Stockholm, Sweden

## Abstract

Glutathione is a tripeptide composed of glutamate, cysteine and glycine. Glutathione is present in millimolar concentrations in most mammalian cells and it is involved in several fundamental biological functions, including free radical scavenging, detoxification of xenobiotics and carcinogens, redox reactions, biosynthesis of DNA, proteins and leukotrienes, as well as neurotransmission/neuromodulation. Glutathione is metabolised *via *the gamma-glutamyl cycle, which is catalyzed by six enzymes. In man, hereditary deficiencies have been found in five of the six enzymes. Glutathione synthetase deficiency is the most frequently recognized disorder and, in its severe form, it is associated with hemolytic anemia, metabolic acidosis, 5-oxoprolinuria, central nervous system (CNS) damage and recurrent bacterial infections. Gamma-glutamylcysteine synthetase deficiency is also associated with hemolytic anemia, and some patients with this disorder show defects of neuromuscular function and generalized aminoaciduria. Gamma-glutamyl transpeptidase deficiency has been found in patients with CNS involvement and glutathionuria. 5-Oxoprolinase deficiency is associated with 5-oxoprolinuria but without a clear association with other symptoms. Dipeptidase deficiency has been described in one patient. All disorders are very rare and inherited in an autosomal recessive manner. Most of the mutations are leaky so that many patients have residual enzyme activity. Diagnosis is made by measuring the concentration of different metabolites in the gamma-glutamyl cycle, enzyme activity and in glutathione synthetase and gamma-glutamylcysteine synthetase deficiency, also by mutation analysis. Prenatal diagnosis has been preformed in glutathione synthetase deficiency. The prognosis is difficult to predict, as few patients are known, but seems to vary significantly between different patients. The aims of the treatment of glutathione synthesis defects are to avoid hemolytic crises and to increase the defense against reactive oxygen species. No treatment has been recommended for gamma-glutamyl transpeptidase, 5-oxoprolinase and dipeptidase deficiency.

## A. Gamma-glutamylcysteine synthetase deficiency

### Disease name and synonyms

Gamma-glutamylcysteine synthetase deficiency (OMIM #230450)

Glutamate-cysteine ligase deficiency

### Definition and diagnostic criteria

Gamma-glutamylcysteine synthetase deficiency is a very rare autosomal recessive disease characterized by hemolytic anemia, and, in some cases, by neurological symptoms. The diagnosis is established by:

• Low activity of gamma-glutamylcysteine synthetase in red blood cells, leukocytes and/or cultured skin fibroblasts.

• Low levels of glutathione and gamma-glutamylcysteine in red blood cells and/or cultured skin fibroblasts.

• Presence of mutation(s) in the gamma-glutamylcysteine synthetase genes. The patients whose results have been published have had homozygous mutations in the gene encoding the heavy subunit of the enzyme.

In red blood cells heterozygous carriers have an enzyme activity of about 50% of the normal mean and normal levels of glutathione [1].

### Epidemiology

Gamma-glutamylcysteine synthetase deficiency is very rare disease. Nine patients in seven families have been reported worldwide (USA, Germany, Japan, The Netherlands, Poland, and Spain).

### Clinical description

All patients with gamma-glutamylcysteine synthetase deficiency have had hemolytic anemia, usually rather mild [1-8]. In addition, two siblings also had spinocerebellar degeneration, peripheral neuropathy, myopathy and aminoaciduria [3,8]. Treatment with sulfonamide precipitated psychosis and pronounced hemolytic anemia in one of these siblings. One patient was reported to have learning disability with dyslexia and was also thought to be mentally retarded [5], and another had delayed psychomotor development and progressive sensory neuropathy of lower extremities, ataxia, hyperreflexia, dysarthria, and a peculiar gait suggestive of spinocerebellar degeneration [2]. Other symptoms found in patients with **γ**-glutamylcysteine synthetase deficiency are transient jaundice, reticulocytosis, and hepatosplenomegaly.

### Etiology

**Figure 1:**
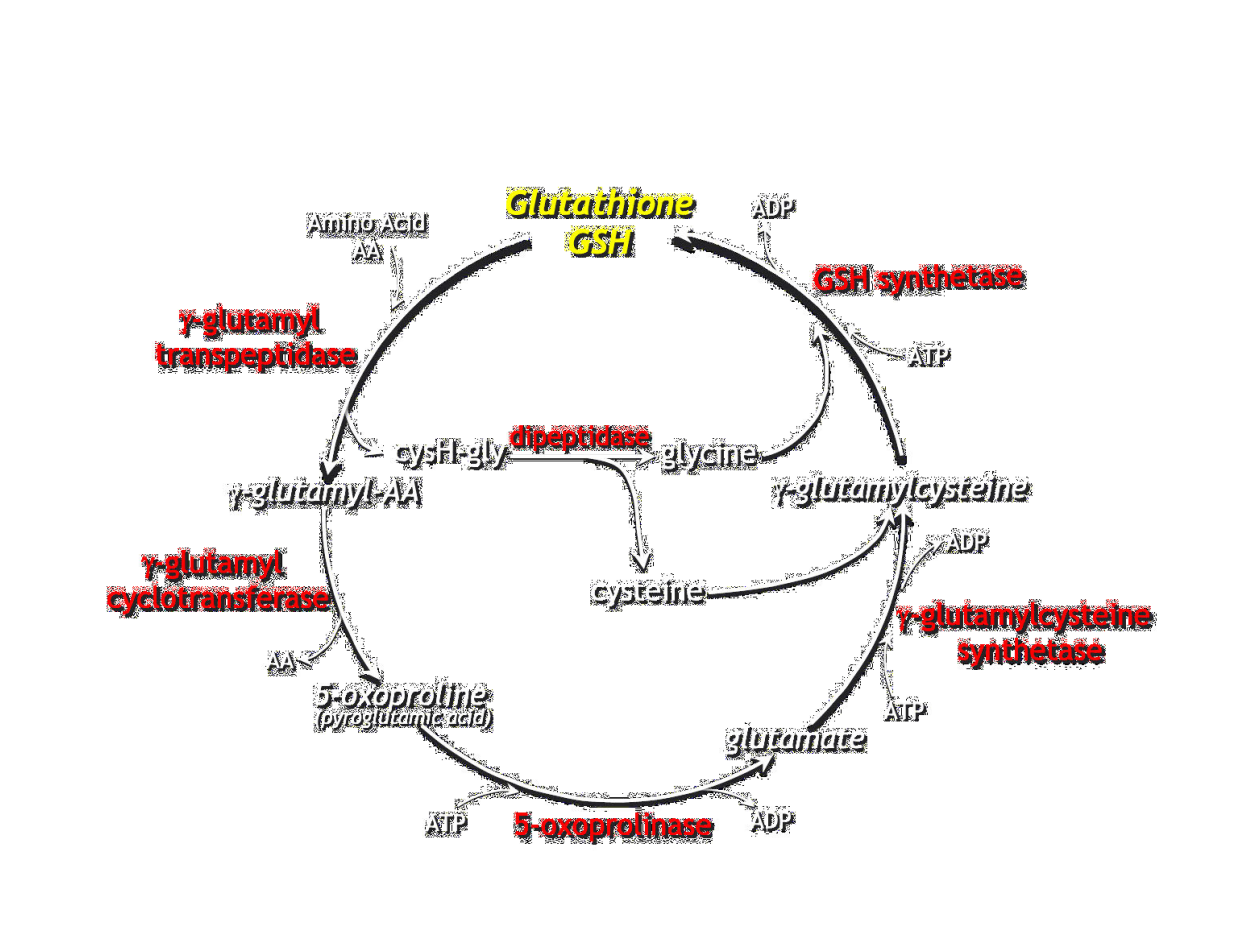
The gamma-glutamyl cycle.

Gamma-glutamylcysteine synthetase catalyzes the first and rate-limiting step in the synthesis of glutathione (GSH) (Figure 1) [9]. Hereditary gamma-glutamylcysteine synthetase deficiency is transmitted as an autosomal recessive trait. The human gamma-glutamylcysteine synthetase enzyme is a dimer consisting of a heavy (catalytic) and a light (regulatory) subunit. The human gene for the heavy subunit has been localized to chromosome 6p12 [10] and the gene for the light subunit to chromosome 1p21 [11]. The heavy subunit (molecular weight about 73 kDa) exhibits the catalytic activity of the native enzyme and is also responsible for the feedback inhibition by GSH. The light subunit (molecular weight about 28 kDa) is catalytically inactive but plays an important regulatory role [12].

Four different mutations in the heavy subunit have been identified in four families affected by gamma-glutamylcysteine synthetase deficiency [1,2,4,5].

Gammaglutamylcysteine synthetase knock-out mice have been developed for both the heavy and the light subunits [13-15]. Homozygous mice embryos with knockout of the heavy subunit fail to gastrulate and die before day 8.5 of gestation [13]. The knockout mice of the light subunit are viable and fertile and have no overt phenotype [15].

### Diagnostic methods

Glutathione and sulphydryl compunds in erythrocytes can be measured using 5,5'-dithiobis (2-nitrobenzoic acid), while glutathione in fibroblasts can be measured with the 5,5'-dithiobis (2-nitrobenzoic acid) glutathione recycling assay [16], or using high performance liquid chromatography (HPLC) [17].

Gamma-glutamylcysteine synthetase in erythrocytes and/or cultured fibroblasts can be measured as described elsewhere [1].

Sequence analysis can be made by polymerase chain reaction (PCR) and sequence analysis of the **γ**-glutamylcysteine synthetase genes (*GLCLC*, *GLCLR*) [1].

### Differential diagnosis

Low levels of GSH can also be due to glutathione synthetase deficiency.

### Genetic counseling

Families with gamma-glutamylcysteine synthetase deficiency can be tested in order to analyze the enzyme activity as well as their mutations. Limited mutation analysis data are available but it is essential to determine the underlying mutations to learn more about the functional properties of the mutant enzyme. Families should also be referred for genetic counseling.

### Antenatal diagnosis

Antenatal diagnosis has not been reported. However, it should be possible if needed by measurement of gamma-glutamylcysteine synthetase activity or mutational analysis (if the mutation in the family is known) of chorionic villi or cultured amniocytes.

### Management including treatment

Patients with gamma-glutamylcysteine synthetase deficiency should avoid drugs known to precipitate hemolytic crises in patients with glucose-6-phosphate dehydrogenase deficiency, *e.g*. phenobarbital, acetylsalicylic acid, sulfonamides. It is possible that patients would benefit from treatment with anti-oxidants but no studies have been made.

## B. Glutathione synthetase deficiency

### Disease name and synonyms

Glutathione synthetase deficiency (OMIM #266130).

5-oxoprolinuria (pyroglutamic aciduria) is sometimes used when referring to glutathione synthetase deficiency; it should be borne in mind however that 5-oxoprolinuria may have other causes (see 'Differential diagnosis').

### Definition and diagnostic criteria

Hereditary glutathione synthetase deficiency is a rare autosomal recessive disease characterized by hemolytic anemia, metabolic acidosis, 5-oxoprolinuria, progressive neurological symptoms and recurrent bacterial infections. The diagnosis usually involves the following: clinical findings, the finding of 5-oxoprolinuria, low levels of glutathione, low activity of glutathione synthetase, and mutation analysis of the glutathione synthetase gene. The diagnosis of glutathione synthetase deficiency is established by:

• Low activity of glutathione synthetase in cultured skin fibroblasts and/or red blood cells.

• Low levels of glutathione in red blood cells and/or cultured skin fibroblasts.

• Urinary 5-oxoproline (up to 1 g/kg/day)

• Mutation(s) in the glutathione synthetase (*GSS*) gene.

Heterozygous carriers have an enzyme activity of about 55% of the normal mean and normal levels of glutathione [18].

### Epidemiology

More than seventy patients have been reported in more than 50 families worldwide.

### Clinical description

According to the clinical symptoms, glutathione synthetase deficiency can be classified as mild, moderate or severe [19]:

#### Mild glutathione synthetase deficiency

These patients show mild hemolytic anemia as their only clinical symptom. In very rare cases, they may excrete excessive amounts of 5-oxoproline in their urine (reference range < 0.1 mol/mol creatinine) but they usually maintain sufficient cellular levels of glutathione to prevent accumulation of 5-oxoproline in body fluids.

#### Moderate glutathione synthetase deficiency

Patients with the moderate variant usually present in the neonatal period with severe and chronic metabolic acidosis, 5-oxoprolinuria, and mild/moderate hemolytic anemia.

#### Severe glutathione synthetase deficiency

Patients have symptoms as in moderate glutathione synthetase deficiency and, in addition, they develop progressive neurological symptoms, *e.g*. psychomotor retardation, mental retardation, seizures, spasticity, ataxia, and intention tremor. Some patients with severe glutathione synthetase deficiency also develop recurrent bacterial infections, probably due to defective granulocyte function. Retinal dystrophy has also been observed in adult patients[20].

Several patients died in early life.

### Etiology

Glutathione synthetase catalyses the last step in the synthesis of glutathione and a deficiency results in low levels of glutathione (Figure 1). Acidosis is due to reduced feedback inhibition of **γ**-glutamyl cysteine synthetase in the gamma-glutamyl cycle, which ultimately leads to overproduction and accumulation of 5-oxoproline. The human glutathione synthetase enzyme is a homodimer with a subunit size of 52 kDa. The three-dimensional structure of the glutathione synthetase enzyme has been established [21]. The gene has been localized to chromosome 20q11.2 and its structure determined [22,23]. Since the human genome contains only one glutathione synthetase gene, the various clinical forms of glutathione synthetase deficiency reflect different mutations or epigenetic modifications in the glutathione synthetase gene. Several mutations have been identified.

The mechanism of metabolic acidosis and 5-oxoprolinuria is the following: decreased levels of cellular glutathione lead to decreased feed-back inhibition of **γ**-glutamylcysteine synthetase. This results in excessive formation of the dipeptide **γ**-glutamylcysteine, which is converted by **γ**-glutamyl cyclotransferase into 5-oxoproline. The overproduction of 5-oxoproline exceeds the capacity of 5-oxoprolinase, and 5-oxoproline therefore accumulates in body fluids and is excreted in the urine.

Patients with glutathione synthetase deficiency accumulate the dipeptide **γ**-glutamylcysteine and cysteine in fibroblasts [24]. As **γ**-glutamylcysteine contains both reactive groups of glutathione (*i.e*. the sulphydryl and **γ**-glutamyl groups) it may to some extent compensate for glutathione in the cellular defence against oxidative stress.

Glutathione is participating in leukotriene C4 (LTC4) synthesis, the primary cysteinyl leukotriene. It has been shown that the synthesis of cysteinyl leukotriens is impaired in patients with glutathione synthetase deficiency [25].

### Diagnostic methods

Urinary 5-oxoproline can be determined by gas chromatography-mass spectrometry [26]. Glutathione and sulphydryls in erythrocytes can be measured using 5,5'-dithiobis (2-nitrobenzoic acid), while glutathione in fibroblasts can be measured with the 5,5'-dithiobis (2-nitrobenzoic acid) glutathione recycling assay [16], or using HPLC [17].

Glutathione synthetase in erythrocytes and/or cultured fibroblasts can be measured as described elsewhere [1].

Mutation analysis of the glutathione synthetase gene (*GSS*) can be made as described by Shi *et al*. 1996 and Njålsson *et al*. 2003 [27,28].

### Differential diagnosis

For other causes of 5-oxoprolinuria, beside glutathione synthetase deficiency and 5-oxoprolinase deficiency [9,29], please see Table 1.

**Table 1:** Causes of 5-oxoprolinuria beside glutathione synthetase deficiency and 5-oxoprolinase deficiency.

**Etiology**	**Possible mechanism**	**Ref.**
Diet	Certain infant formulas and tomato juice may contain proteins modified by preparation that have increased 5-oxoproline content	[52]
Severe burns Stevens-Johnson syndrome	Increased metabolism of collagen, fibrinogen or other proteins that contain substantial amounts of 5-oxoproline	[53]
Other inborn errors of metabolism	Inborn errors of metabolism not involving the gamma-glutamyl cycle, e.g. X-linked ornithine trancarbamylase deficiency, urea cycle defects, tyrosinemia. In critical organs (e.g. liver, kidney), lack of ATP, which is needed for conversion of 5-oxoproline into glutamate, may lead to 5-oxoprolinuria	[29] [9]
Homocystinuria	Patients with homocystinuria may have excessive formation of 5-oxoproline	[54]
Drug metabolism	Paracetamol, vigabatrin and antibiotics (flucloxacillin, netimicin) probably interact with the gamma-glutamyl cycle	[55-57]
Prematurity	Transient 5-oxoprolinuria has been observed in very preterm infants. The cause is unknown	[58] [29, 59]
Malnutrition, pregnancy	Limited availability of glycine	[60]
Nephropatic cystinosis	Nephropatic cystinosis patients: may have 5-oxoprolinuria probably because of decreased availability of free cysteine, resulting in a secondary impairment of the **γ**-glutamyl cycle. Cysteamine therapy normalizes the 5-oxoprolinuria	[61]

Genetic counseling

Families with glutathione synthetase deficiency can be referred for testing in order to analyze the enzyme activity as well as their mutations. It is essential to find out whether it is possible to correlate the genotype with the phenotype.

### Antenatal diagnosis

Antenatal diagnosis is possible and can be made by:

• Mutation analysis of chorionic villi (the method of choice if the mutation in the family is known).

• Analysis of 5-oxoproline in amniotic fluid [30,31].

• Analysis of glutathione synthetase activity in cultured amniocytes or chorionic villi [30].

### Management including treatment

The clinical goals of management are correction of acidosis and early supplementation with vitamin C (ascorbic acid) and vitamin E (alpha-tocopherol) [19]. The acidosis is corrected with bicarbonate. The recommended dose of vitamin C is 100 mg/kg/day and of vitamin E 10 mg/kg/day. N-Acetylcysteine used to be recommended because it may protect cells from oxidative stress. However, cysteine has been shown to accumulate in tissues of patients with glutathione synthetase deficiency – at least in cultured fibroblasts [24]. Since cysteine is known to be neurotoxic in excessive amounts [32], treatment with N-acetylcysteine should not be recommended for patients with glutathione synthetase deficiency as this may increase the intracellular cysteine levels even more.

Patients with glutathione synthetase deficiency should avoid drugs known to precipitate hemolytic crises in patients with glucose-6-phosphate dehydrogenase deficiency.

### Prognosis

A long-term follow up study of 28 patients with glutathione synthetase deficiency has showed that the factors most predictive of survival and long-term outcome are early diagnosis, correction of acidosis and early supplementation with vitamin C and vitamin E [19].

## C. Gamma-glutamyl transpeptidase deficiency

### Disease name

Gamma-glutamyl transpeptidase deficiency (OMIM 231950).

### Definition and diagnostic criteria

Gamma-glutamyl transpeptidase deficiency is a very rare autosomal recessive disease characterized by increased glutathione concentration in plasma and urine. Central nervous system involvement may also be present. The diagnosis is established by:

• Low activity of **γ**-glutamyl transpeptidase in nucleated cells such as leukocytes or cultured skin fibroblasts. Erythrocytes also lack **γ**-glutamyl transpeptidase under normal conditions.

• High levels of glutathione in plasma and urine (up to 1 g/day in urine; controls < 10 mg). Cellular levels of glutathione are normal.

### Epidemiology

Gamma-glutamyl transpeptidase deficiency is a very rare disease, which has been reported in seven patients in five families worldwide [33-37].

### Clinical description

Five out of seven reported patients had central nervous system involvement [33-37]. Whether these symptoms are part of the clinical picture remains to be established. All patients have had glutathionuria (up to 1 g/day; controls < 10 mg). In addition, patients have increased urinary levels of gamma-glutamylcysteine and cysteine. Three patients with **γ**-glutamyl transpeptidase deficiency have been studied and found to have a complete deficiency of leukotriene D4 biosynthetsis [38].

### Etiology

The human gamma-glutamyl transpeptidase gene family is composed of at least seven different gene loci and several of them are located on the long arm of chromosome 22 [9]. Gamma-glutamyl transpeptidase is a heterodimer with subunits of 21 kDa and 38 kDa. The enzyme is membrane-bound with its active site facing the external side of the cell. Erythrocytes lack gamma-glutamyl transpeptidase and this enzyme activity also varies in other tissues. Gamma-glutamyl transpeptidase catalyses the first step in the degradation of glutathione (Figure 1). No mutations have been identified in patients with gamma-glutamyl transpeptidase deficiency. A knock-out mouse for **γ**-glutamyl transpeptidase has been developed. Gamma-glutamyl transpeptidase deficiency leads to glutathionuria, glutathionemia, growth failure, cataracts, lethargy, shortened life span, and infertility. A closer study of the reproductive phenotype of **γ**-glutamyl transpeptidase deficient mice show that they are hypogonadal and infertile [39].

### Diagnostic methods

Glutathione in plasma and urine can be determined by various chromatographic or calorimetric techniques.

Gamma-glutamyl transpeptidase in nucleated cells can be determined using the method described by Wright *et al*. [36].

### Genetic counseling

The disease is transmitted as an autosomal recessive trait. Patients should be offered genetic counseling.

### Antenatal diagnosis

Antenatal diagnosis has not been reported.

### Management including treatment

No specific treatment has been proposed or tried. However, administration of N-acetylcysteine to **γ**-glutamyl transpeptidase deficient mutant mice for two weeks restored their fertility [39].

## D. 5-Oxoprolinase deficiency

Disease name

5-Oxoprolinase deficiency (OMIM 260005)

### Definition and diagnostic criteria

5-Oxoprolinase deficiency is a very rare autosomal recessive disease characterized by 5-oxoprolinuria and very heterogeneous clinical presentation (renal stone formation, enterocolitis, mental retardation, neonatal hypoglycemia, microcytic anemia and microcephaly).

The diagnosis of 5-oxoprolinase deficiency is established by:

• Low activity of 5-oxoprolinase in nucleated cells such as leukocytes or cultured skin fibroblasts (5-oxoprolinase is not present in erythrocytes).

• Elevated levels of 5-oxoproline in body fluids.

• Urinary 5-oxoproline.

### Epidemiology

Eight patients have been reported worldwide.

### Clinical description

All patients with 5-oxoprolinase deficiency have been identified because of 5-oxoprolinuria (4 to 10 g/day. Reference range < 0.1 mol/mol creatinine), but they lack a consistent clinical picture [9]. They have normal acid-base balance. Different clinical symptoms reported in individual patients with 5-oxoprolinase deficiency are renal stone formation, enterocolitis, mental retardation, neonatal hypoglycemia, microcytic anemia and microcephaly [40-45].

### Etiology

5-Oxoprolinase catalyses a step in the gamma-glutamyl cycle (glutathione metabolism), the ring-opening of 5-oxoproline to yield glutamate. 5-Oxoprolinase is the enzyme in the gamma-glutamyl cycle with the lowest capacity (Figure 1).

The mammalian enzyme is not well studied, but it is apparently composed of two identical subunits. The mechanism leading to 5-oxoprolinuria is the following: decreased activity of 5-oxoprolinase leads to decreased conversion of 5-oxoproline to glutamate. Therefore, 5-oxoproline accumulates in body fluids and is excreted in the urine. The quantities are less than those found in patients with glutathione synthetase deficiency and therefore acid-base balance is usually normal.

### Diagnostic methods

The level of 5-oxoproline in urine can be determined by gas chromatography-mass spectrometry [26]. Activity of 5-oxoprolinase in leukocytes and/or cultured fibroblasts can be measured according to the method described by Larsson *et al*. [46].

### Differential diagnosis

For other causes of 5-oxoprolinuria, besides 5-oxoprolinase deficiency and glutathione synthetase deficiency [9,29], please see Table 1.

### Genetic counseling

Families with 5-oxoprolinase deficiency can be tested in order to analyze the enzyme activity. They should be referred for genetic counseling.

### Antenatal diagnosis

Antenatal diagnosis of 5-oxoprolinase deficiency has not been reported to date.

### Management including treatment

No specific treatment has been proposed or tried.

## E. Dipeptidase deficiency

### Disease name and synonyms

Dipeptidase deficiency

Cysteinylglycinuria

Cysteineglycinas deficiency

### Definition and diagnostic criteria

Dipeptidase deficiency is an extremely rare diseases autosomal recessive manner, characterized by increased urinary excretion of cysteinylglycine and a pathological excretion pattern of leukotriens. The diagnosis is based on the finding of cysteinylglycinuria and decreased activity of dipeptidase, and is established by:

• Increased urinary excretion of cysteinyl glycine

• Normal concentration of cysteinyl glycine in plasma

• Low activity of dipeptidase in cultured skin fibroblasts and/or red blood cells.

### Epidemiology

Dipeptidase deficiency has been suggested in only one patient worldwide. The diagnosis has not been confirmed by enzyme analysis in this patient.

### Clinical description

One patient with a suspected membrane-bound dipeptidase deficiency has been described [47]. This was a 15-year-old boy who presented with mental retardation, mild motor impairment, and partial deafness. Biochemical investigations showed a normal level of cysteinyl glycine in plasma, an abnormal urinary profile with increased excretion of cysteinyl glycine (4972 mmol/mol of creatinine) and leukotrienes with increased leukotriene D4 and complete absence of LTE4. The concentration of leukotriene D(4) (LTD(4)), which is usually not detectable, was highly increased, whereas LTE(4), the major urinary metabolite in humans, was completely absent. These data suggest membrane-bound dipeptidase deficiency [47].

### Etiology

Membrane-bound dipeptidase (E.C. 3.4.13.19) is the enzyme that hydrolyzes dipeptides, including cysteinylglycine compounds, such as the oxidized **γ**-glutamyltranspeptidase product cystinyl-bis-glycine and the conversion of leukotriene D4 to E4 [48]. Dipeptidase also hydrolyzes certain **β**-lactam antibiotics. The protein is 42 kDa unglycosylated and 63 kDa when glycosylated. The crystal structure of human membrane-bound dipeptidase has been reported [49]. Renal dipeptidase has been mapped to human chromosome 16 at q24 [50]. A deficiency of dipeptidase has been suspected in only one patient.

### Diagnostic methods

The diagnosis is made by quantitative amino acid analysis of urine.

The activity of dipeptidase can be assayed with a HPLC method using glycyl-D-phenylalanine as described by Littlewood *et al*. 1989 [51]. To date, the corresponding enzyme deficiency has not been assayed in the patient with suspected dipeptidase deficiency.

### Genetic counseling

Patients should be offered genetic counseling.

### Antenatal diagnosis

Not relevant.

### Management including treatment

No specific treatment has been proposed or tried.

### Unresolved questions

• It remains to be established whether CNS symptoms reported in some patients with gamma-glutamylcysteine synthetase are related to the enzyme defect or not. Also, strategies for treatment need to be investigated.

• It remains to be established if free radicals are involved in the pathogenesis of glutathione synthetase deficiency and if patients with low levels of glutathione are more sensitive to oxidative stress.

• The relationship between genotype and phenotype in gamma-glutamyl transpeptidase deficiency remains to be established. Are the symptoms in the identified patients merely a coincidence?

• The relationship between genotype and phenotype in 5-oxoprolinase deficiency remains to be established. It is unknown whether symptoms in identified patients are merely a coincidence.

• In dipeptidase deficiency, it remains to be established if there is a relationship between the biochemical defect and the clinical symptoms.

• Prognosis of gamma-glutamylcysteine synthetase deficiency, gamma-glutamyl transpeptidase deficiency, 5-oxoprolinase deficiency and dipeptidase deficiency is unclear and difficult to predict as very few patients have been reported worldwide.
